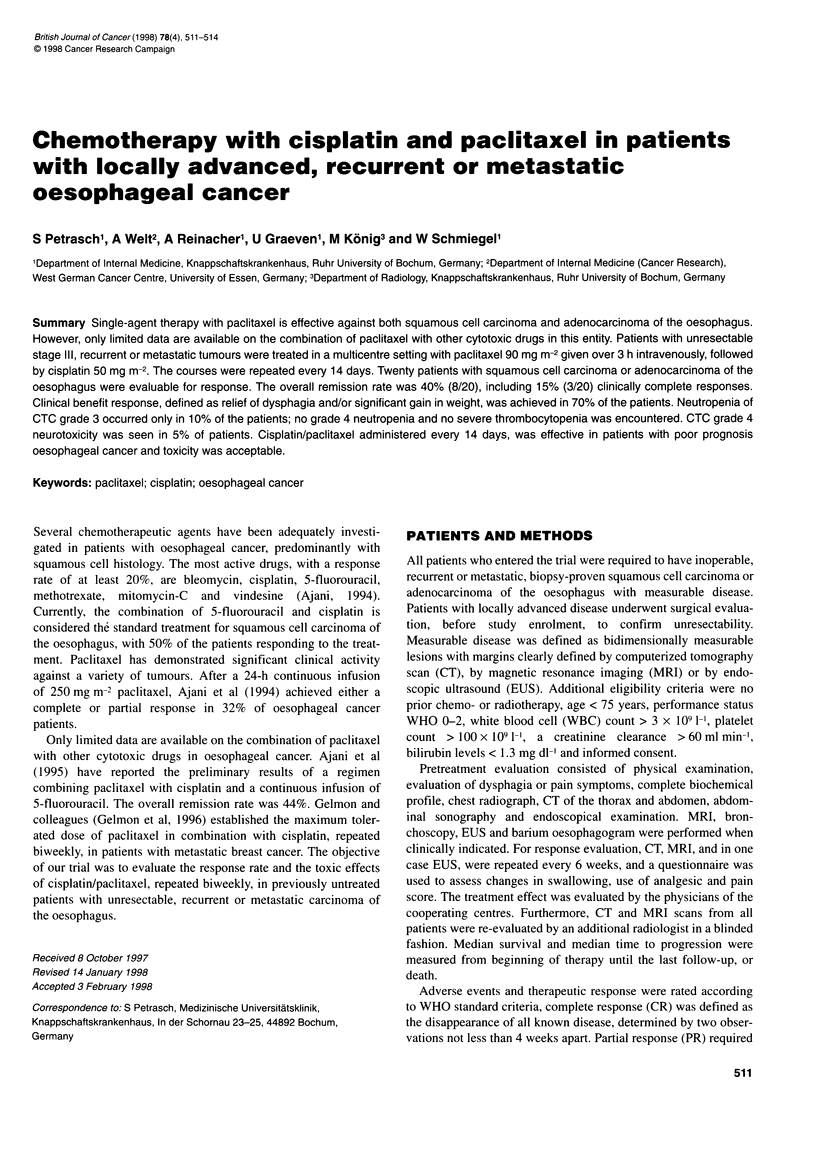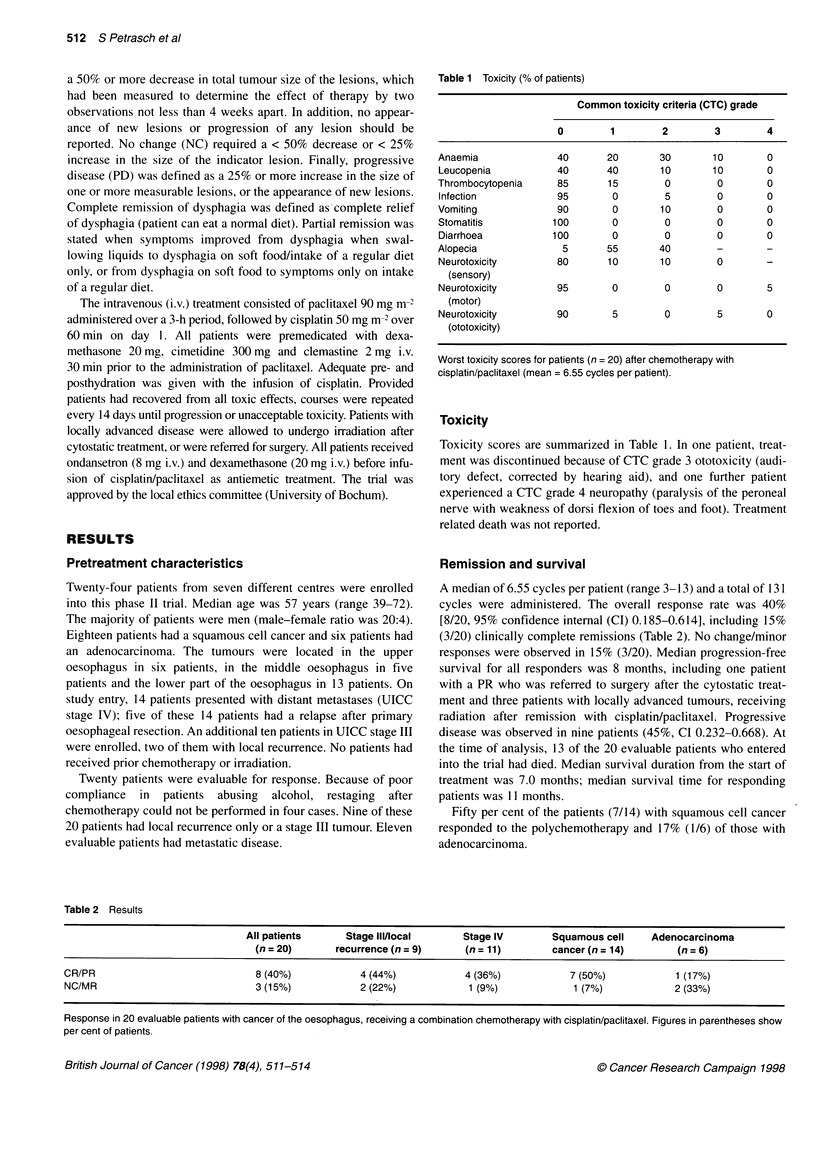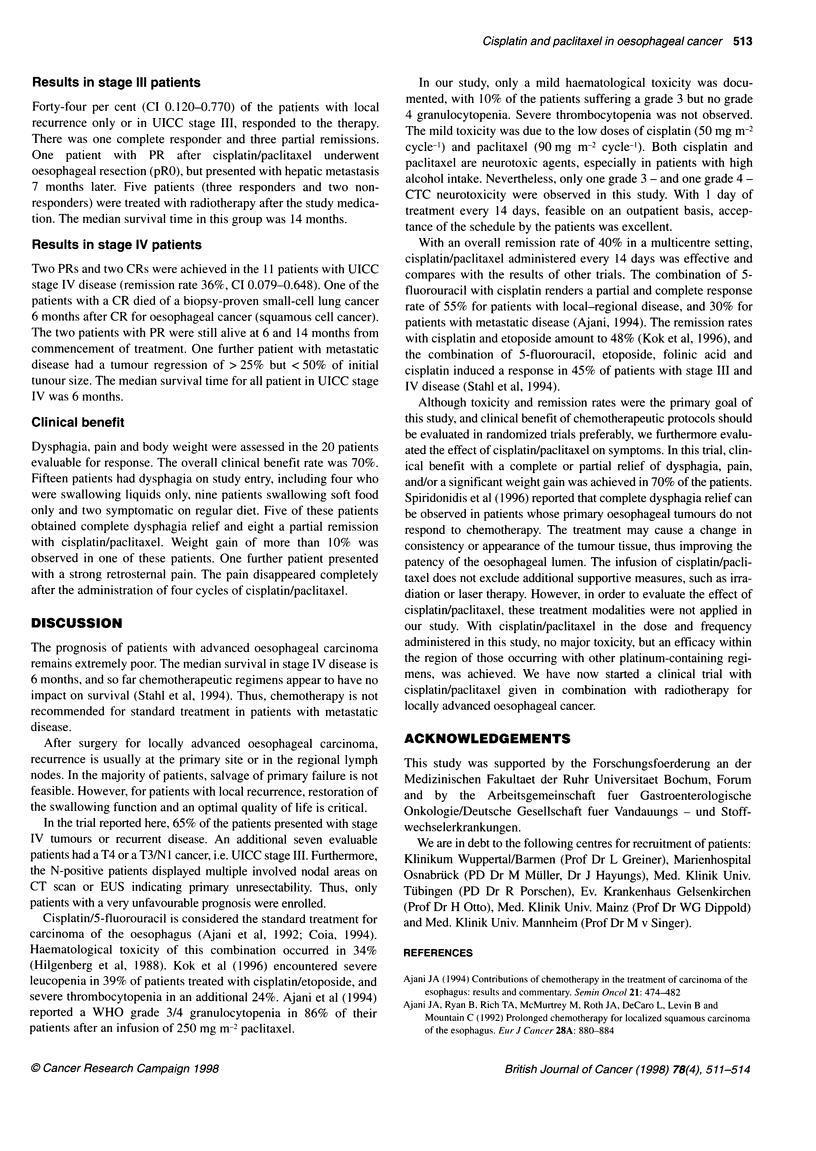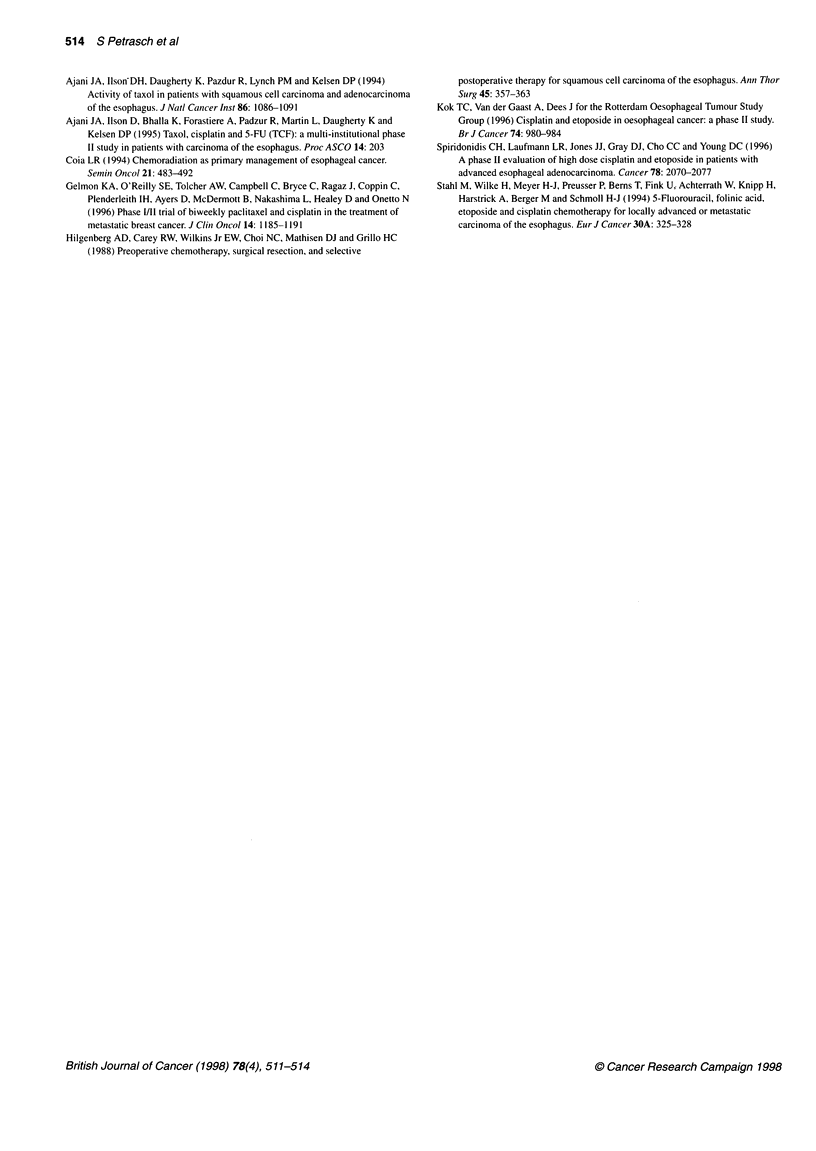# Chemotherapy with cisplatin and paclitaxel in patients with locally advanced, recurrent or metastatic oesophageal cancer.

**DOI:** 10.1038/bjc.1998.524

**Published:** 1998-08

**Authors:** S. Petrasch, A. Welt, A. Reinacher, U. Graeven, M. König, W. Schmiegel

**Affiliations:** Department of Internal Medicine, Knappschaftskrankenhaus, Ruhr University of Bochum, Germany.

## Abstract

Single-agent therapy with paclitaxel is effective against both squamous cell carcinoma and adenocarcinoma of the oesophagus. However, only limited data are available on the combination of paclitaxel with other cytotoxic drugs in this entity. Patients with unresectable stage III, recurrent or metastatic tumours were treated in a multicentre setting with paclitaxel 90 mg m(-2) given over 3 h intravenously, followed by cisplatin 50 mg m(-2). The courses were repeated every 14 days. Twenty patients with squamous cell carcinoma or adenocarcinoma of the oesophagus were evaluable for response. The overall remission rate was 40% (8/20), including 15% (3/20) clinically complete responses. Clinical benefit response, defined as relief of dysphagia and/or significant gain in weight, was achieved in 70% of the patients. Neutropenia of CTC grade 3 occurred only in 10% of the patients; no grade 4 neutropenia and no severe thrombocytopenia was encountered. CTC grade 4 neurotoxicity was seen in 5% of patients. Cisplatin/paclitaxel administered every 14 days, was effective in patients with poor prognosis oesophageal cancer and toxicity was acceptable.


British Journal of Cancer (1998) 78(4), 511–514
© 1998 Cancer Research Campaign

### Chemotherapy with cisplatin and paclitaxel in patients with locally advanced, recurrent or metastatic oesophageal cancer

**S Petrasch[1], A Welt[2], A Reinacher[1], U Graeven[1], M König[3] and W Schmiegel[1]**

[1]Department of Internal Medicine, Knappschaftskrankenhaus, Ruhr University of Bochum, Germany; [2]Department of Internal Medicine (Cancer Research), West German Cancer Centre, University of Essen, Germany; [3]Department of Radiology, Knappschaftskrankenhaus, Ruhr University of Bochum, Germany

**Summary** Single-agent therapy with paclitaxel is effective against both squamous cell carcinoma and adenocarcinoma of the oesophagus. However, only limited data are available on the combination of paclitaxel with other cytotoxic drugs in this entity. Patients with unresectable stage III, recurrent or metastatic tumours were treated in a multicentre setting with paclitaxel 90 mg $m^{-2}$ given over 3 h intravenously, followed by cisplatin 50 mg $m^{-2}$. The courses were repeated every 14 days. Twenty patients with squamous cell carcinoma or adenocarcinoma of the oesophagus were evaluable for response. The overall remission rate was 40% (8/20), including 15% (3/20) clinically complete responses. Clinical benefit response, defined as relief of dysphagia and/or significant gain in weight, was achieved in 70% of the patients. Neutropenia of CTC grade 3 occurred only in 10% of the patients; no grade 4 neutropenia and no severe thrombocytopenia was encountered. CTC grade 4 neurotoxicity was seen in 5% of patients. Cisplatin/paclitaxel administered every 14 days, was effective in patients with poor prognosis oesophageal cancer and toxicity was acceptable.

**Keywords:** paclitaxel; cisplatin; oesophageal cancer

Several chemotherapeutic agents have been adequately investigated in patients with oesophageal cancer, predominantly with squamous cell histology. The most active drugs, with a response rate of at least 20%, are bleomycin, cisplatin, 5-fluorouracil, methotrexate, mitomycin-C and vindesine (Ajani, 1994). Currently, the combination of 5-fluorouracil and cisplatin is considered the standard treatment for squamous cell carcinoma of the oesophagus, with 50% of the patients responding to the treatment. Paclitaxel has demonstrated significant clinical activity against a variety of tumours. After a 24-h continuous infusion of 250 mg $m^{-2}$ paclitaxel, Ajani et al (1994) achieved either a complete or partial response in 32% of oesophageal cancer patients.

Only limited data are available on the combination of paclitaxel with other cytotoxic drugs in oesophageal cancer. Ajani et al (1995) have reported the preliminary results of a regimen combining paclitaxel with cisplatin and a continuous infusion of 5-fluorouracil. The overall remission rate was 44%. Gelmon and colleagues (Gelmon et al, 1996) established the maximum tolerated dose of paclitaxel in combination with cisplatin, repeated biweekly, in patients with metastatic breast cancer. The objective of our trial was to evaluate the response rate and the toxic effects of cisplatin/paclitaxel, repeated biweekly, in previously untreated patients with unresectable, recurrent or metastatic carcinoma of the oesophagus.

*Received 8 October 1997*
*Revised 14 January 1998*
*Accepted 3 February 1998*

*Correspondence to:* S Petrasch, Medizinische Universitätsklinik, Knappschaftskrankenhaus, In der Schornau 23–25, 44892 Bochum, Germany

### PATIENTS AND METHODS

All patients who entered the trial were required to have inoperable, recurrent or metastatic, biopsy-proven squamous cell carcinoma or adenocarcinoma of the oesophagus with measurable disease. Patients with locally advanced disease underwent surgical evaluation, before study enrolment, to confirm unresectability. Measurable disease was defined as bidimensionally measurable lesions with margins clearly defined by computerized tomography scan (CT), by magnetic resonance imaging (MRI) or by endoscopic ultrasound (EUS). Additional eligibility criteria were no prior chemo- or radiotherapy, age < 75 years, performance status WHO 0–2, white blood cell (WBC) count $> 3 \times 10^9$ $l^{-1}$, platelet count $> 100 \times 10^9$ $l^{-1}$, a creatinine clearance > 60 ml $min^{-1}$, bilirubin levels < 1.3 mg $dl^{-1}$ and informed consent.

Pretreatment evaluation consisted of physical examination, evaluation of dysphagia or pain symptoms, complete biochemical profile, chest radiograph, CT of the thorax and abdomen, abdominal sonography and endoscopical examination. MRI, bronchoscopy, EUS and barium oesophagogram were performed when clinically indicated. For response evaluation, CT, MRI, and in one case EUS, were repeated every 6 weeks, and a questionnaire was used to assess changes in swallowing, use of analgesic and pain score. The treatment effect was evaluated by the physicians of the cooperating centres. Furthermore, CT and MRI scans from all patients were re-evaluated by an additional radiologist in a blinded fashion. Median survival and median time to progression were measured from beginning of therapy until the last follow-up, or death.

Adverse events and therapeutic response were rated according to WHO standard criteria, complete response (CR) was defined as the disappearance of all known disease, determined by two observations not less than 4 weeks apart. Partial response (PR) required a 50% or more decrease in total tumour size of the lesions, which had been measured to determine the effect of therapy by two observations not less than 4 weeks apart. In addition, no appearance of new lesions or progression of any lesion should be reported. No change (NC) required a < 50% decrease or < 25% increase in the size of the indicator lesion. Finally, progressive disease (PD) was defined as a 25% or more increase in the size of one or more measurable lesions, or the appearance of new lesions. Complete remission of dysphagia was defined as complete relief of dysphagia (patient can eat a normal diet). Partial remission was stated when symptoms improved from dysphagia when swallowing liquids to dysphagia on soft food/intake of a regular diet only, or from dysphagia on soft food to symptoms only on intake of a regular diet.

The intravenous (i.v.) treatment consisted of paclitaxel 90 mg $m^{-2}$ administered over a 3-h period, followed by cisplatin 50 mg $m^{-2}$ over 60 min on day 1. All patients were premedicated with dexamethasone 20 mg, cimetidine 300 mg and clemastine 2 mg i.v. 30 min prior to the administration of paclitaxel. Adequate pre- and posthydration was given with the infusion of cisplatin. Provided patients had recovered from all toxic effects, courses were repeated every 14 days until progression or unacceptable toxicity. Patients with locally advanced disease were allowed to undergo irradiation after cytostatic treatment, or were referred for surgery. All patients received ondansetron (8 mg i.v.) and dexamethasone (20 mg i.v.) before infusion of cisplatin/paclitaxel as antiemetic treatment. The trial was approved by the local ethics committee (University of Bochum).

### RESULTS

### Pretreatment characteristics

Twenty-four patients from seven different centres were enrolled into this phase II trial. Median age was 57 years (range 39–72). The majority of patients were men (male–female ratio was 20:4). Eighteen patients had a squamous cell cancer and six patients had an adenocarcinoma. The tumours were located in the upper oesophagus in six patients, in the middle oesophagus in five patients and the lower part of the oesophagus in 13 patients. On study entry, 14 patients presented with distant metastases (UICC stage IV); five of these 14 patients had a relapse after primary oesophageal resection. An additional ten patients in UICC stage III were enrolled, two of them with local recurrence. No patients had received prior chemotherapy or irradiation.

Twenty patients were evaluable for response. Because of poor compliance in patients abusing alcohol, restaging after chemotherapy could not be performed in four cases. Nine of these 20 patients had local recurrence only or a stage III tumour. Eleven evaluable patients had metastatic disease.

**Table 1** Toxicity (% of patients)

| | Common toxicity criteria (CTC) grade | | | | |
|---|---|---|---|---|---|
| | 0 | 1 | 2 | 3 | 4 |
| Anaemia | 40 | 20 | 30 | 10 | 0 |
| Leucopenia | 40 | 40 | 10 | 10 | 0 |
| Thrombocytopenia | 85 | 15 | 0 | 0 | 0 |
| Infection | 95 | 0 | 5 | 0 | 0 |
| Vomiting | 90 | 0 | 10 | 0 | 0 |
| Stomatitis | 100 | 0 | 0 | 0 | 0 |
| Diarrhoea | 100 | 0 | 0 | 0 | 0 |
| Alopecia | 5 | 55 | 40 | – | – |
| Neurotoxicity (sensory) | 80 | 10 | 10 | 0 | – |
| Neurotoxicity (motor) | 95 | 0 | 0 | 0 | 5 |
| Neurotoxicity (ototoxicity) | 90 | 5 | 0 | 5 | 0 |

Worst toxicity scores for patients (*n* = 20) after chemotherapy with cisplatin/paclitaxel (mean = 6.55 cycles per patient).

### Toxicity

Toxicity scores are summarized in Table 1. In one patient, treatment was discontinued because of CTC grade 3 ototoxicity (auditory defect, corrected by hearing aid), and one further patient experienced a CTC grade 4 neuropathy (paralysis of the peroneal nerve with weakness of dorsi flexion of toes and foot). Treatment related death was not reported.

### Remission and survival

A median of 6.55 cycles per patient (range 3–13) and a total of 131 cycles were administered. The overall response rate was 40% [8/20, 95% confidence internal (CI) 0.185–0.614], including 15% (3/20) clinically complete remissions (Table 2). No change/minor responses were observed in 15% (3/20). Median progression-free survival for all responders was 8 months, including one patient with a PR who was referred to surgery after the cytostatic treatment and three patients with locally advanced tumours, receiving radiation after remission with cisplatin/paclitaxel. Progressive disease was observed in nine patients (45%, CI 0.232–0.668). At the time of analysis, 13 of the 20 evaluable patients who entered into the trial had died. Median survival duration from the start of treatment was 7.0 months; median survival time for responding patients was 11 months.

Fifty per cent of the patients (7/14) with squamous cell cancer responded to the polychemotherapy and 17% (1/6) of those with adenocarcinoma.

**Table 2** Results

| | All patients (*n* = 20) | Stage III/local recurrence (*n* = 9) | Stage IV (*n* = 11) | Squamous cell cancer (*n* = 14) | Adenocarcinoma (*n* = 6) |
|---|---|---|---|---|---|
| CR/PR | 8 (40%) | 4 (44%) | 4 (36%) | 7 (50%) | 1 (17%) |
| NC/MR | 3 (15%) | 2 (22%) | 1 (9%) | 1 (7%) | 2 (33%) |

Response in 20 evaluable patients with cancer of the oesophagus, receiving a combination chemotherapy with cisplatin/paclitaxel. Figures in parentheses show per cent of patients.

© Cancer Research Campaign 1998

### Results in stage III patients

Forty-four per cent (CI 0.120–0.770) of the patients with local recurrence only or in UICC stage III, responded to the therapy. There was one complete responder and three partial remissions. One patient with PR after cisplatin/paclitaxel underwent oesophageal resection (pR0), but presented with hepatic metastasis 7 months later. Five patients (three responders and two non-responders) were treated with radiotherapy after the study medication. The median survival time in this group was 14 months.

### Results in stage IV patients

Two PRs and two CRs were achieved in the 11 patients with UICC stage IV disease (remission rate 36%, CI 0.079–0.648). One of the patients with a CR died of a biopsy-proven small-cell lung cancer 6 months after CR for oesophageal cancer (squamous cell cancer). The two patients with PR were still alive at 6 and 14 months from commencement of treatment. One further patient with metastatic disease had a tumour regression of > 25% but < 50% of initial tunour size. The median survival time for all patient in UICC stage IV was 6 months.

### Clinical benefit

Dysphagia, pain and body weight were assessed in the 20 patients evaluable for response. The overall clinical benefit rate was 70%. Fifteen patients had dysphagia on study entry, including four who were swallowing liquids only, nine patients swallowing soft food only and two symptomatic on regular diet. Five of these patients obtained complete dysphagia relief and eight a partial remission with cisplatin/paclitaxel. Weight gain of more than 10% was observed in one of these patients. One further patient presented with a strong retrosternal pain. The pain disappeared completely after the administration of four cycles of cisplatin/paclitaxel.

### DISCUSSION

The prognosis of patients with advanced oesophageal carcinoma remains extremely poor. The median survival in stage IV disease is 6 months, and so far chemotherapeutic regimens appear to have no impact on survival (Stahl et al, 1994). Thus, chemotherapy is not recommended for standard treatment in patients with metastatic disease.

After surgery for locally advanced oesophageal carcinoma, recurrence is usually at the primary site or in the regional lymph nodes. In the majority of patients, salvage of primary failure is not feasible. However, for patients with local recurrence, restoration of the swallowing function and an optimal quality of life is critical.

In the trial reported here, 65% of the patients presented with stage IV tumours or recurrent disease. An additional seven evaluable patients had a T4 or a T3/N1 cancer, i.e. UICC stage III. Furthermore, the N-positive patients displayed multiple involved nodal areas on CT scan or EUS indicating primary unresectability. Thus, only patients with a very unfavourable prognosis were enrolled.

Cisplatin/5-fluorouracil is considered the standard treatment for carcinoma of the oesophagus (Ajani et al, 1992; Coia, 1994). Haematological toxicity of this combination occurred in 34% (Hilgenberg et al, 1988). Kok et al (1996) encountered severe leucopenia in 39% of patients treated with cisplatin/etoposide, and severe thrombocytopenia in an additional 24%. Ajani et al (1994) reported a WHO grade 3/4 granulocytopenia in 86% of their patients after an infusion of 250 mg $m^{-2}$ paclitaxel.

In our study, only a mild haematological toxicity was documented, with 10% of the patients suffering a grade 3 but no grade 4 granulocytopenia. Severe thrombocytopenia was not observed. The mild toxicity was due to the low doses of cisplatin (50 mg $m^{-2}$ $cycle^{-1}$) and paclitaxel (90 mg $m^{-2}$ $cycle^{-1}$). Both cisplatin and paclitaxel are neurotoxic agents, especially in patients with high alcohol intake. Nevertheless, only one grade 3 – and one grade 4 – CTC neurotoxicity were observed in this study. With 1 day of treatment every 14 days, feasible on an outpatient basis, acceptance of the schedule by the patients was excellent.

With an overall remission rate of 40% in a multicentre setting, cisplatin/paclitaxel administered every 14 days was effective and compares with the results of other trials. The combination of 5-fluorouracil with cisplatin renders a partial and complete response rate of 55% for patients with local–regional disease, and 30% for patients with metastatic disease (Ajani, 1994). The remission rates with cisplatin and etoposide amount to 48% (Kok et al, 1996), and the combination of 5-fluorouracil, etoposide, folinic acid and cisplatin induced a response in 45% of patients with stage III and IV disease (Stahl et al, 1994).

Although toxicity and remission rates were the primary goal of this study, and clinical benefit of chemotherapeutic protocols should be evaluated in randomized trials preferably, we furthermore evaluated the effect of cisplatin/paclitaxel on symptoms. In this trial, clinical benefit with a complete or partial relief of dysphagia, pain, and/or a significant weight gain was achieved in 70% of the patients. Spiridonidis et al (1996) reported that complete dysphagia relief can be observed in patients whose primary oesophageal tumours do not respond to chemotherapy. The treatment may cause a change in consistency or appearance of the tumour tissue, thus improving the patency of the oesophageal lumen. The infusion of cisplatin/paclitaxel does not exclude additional supportive measures, such as irradiation or laser therapy. However, in order to evaluate the effect of cisplatin/paclitaxel, these treatment modalities were not applied in our study. With cisplatin/paclitaxel in the dose and frequency administered in this study, no major toxicity, but an efficacy within the region of those occurring with other platinum-containing regimens, was achieved. We have now started a clinical trial with cisplatin/paclitaxel given in combination with radiotherapy for locally advanced oesophageal cancer.

### ACKNOWLEDGEMENTS

This study was supported by the Forschungsfoerderung an der Medizinischen Fakultaet der Ruhr Universitaet Bochum, Forum and by the Arbeitsgemeinschaft fuer Gastroenterologische Onkologie/Deutsche Gesellschaft fuer Vandauungs – und Stoffwechselerkrankungen.

We are in debt to the following centres for recruitment of patients: Klinikum Wuppertal/Barmen (Prof Dr L Greiner), Marienhospital Osnabrück (PD Dr M Müller, Dr J Hayungs), Med. Klinik Univ. Tübingen (PD Dr R Porschen), Ev. Krankenhaus Gelsenkirchen (Prof Dr H Otto), Med. Klinik Univ. Mainz (Prof Dr WG Dippold) and Med. Klinik Univ. Mannheim (Prof Dr M v Singer).

### REFERENCES

Ajani JA (1994) Contributions of chemotherapy in the treatment of carcinoma of the esophagus: results and commentary. *Semin Oncol* **21**: 474–482

Ajani JA, Ryan B, Rich TA, McMurtrey M, Roth JA, DeCaro L, Levin B and Mountain C (1992) Prolonged chemotherapy for localized squamous carcinoma of the esophagus. *Eur J Cancer* **28A**: 880–884

© Cancer Research Campaign 1998

Ajani JA, Ilson DH, Daugherty K, Pazdur R, Lynch PM and Kelsen DP (1994) Activity of taxol in patients with squamous cell carcinoma and adenocarcinoma of the esophagus. *J Natl Cancer Inst* **86**: 1086–1091

Ajani JA, Ilson D, Bhalla K, Forastiere A, Padzur R, Martin L, Daugherty K and Kelsen DP (1995) Taxol, cisplatin and 5-FU (TCF): a multi-institutional phase II study in patients with carcinoma of the esophagus. *Proc ASCO* **14**: 203

Coia LR (1994) Chemoradiation as primary management of esophageal cancer. *Semin Oncol* **21**: 483–492

Gelmon KA, O'Reilly SE, Tolcher AW, Campbell C, Bryce C, Ragaz J, Coppin C, Plenderleith IH, Ayers D, McDermott B, Nakashima L, Healey D and Onetto N (1996) Phase I/II trial of biweekly paclitaxel and cisplatin in the treatment of metastatic breast cancer. *J Clin Oncol* **14**: 1185–1191

Hilgenberg AD, Carey RW, Wilkins Jr EW, Choi NC, Mathisen DJ and Grillo HC (1988) Preoperative chemotherapy, surgical resection, and selective postoperative therapy for squamous cell carcinoma of the esophagus. *Ann Thor Surg* **45**: 357–363

Kok TC, Van der Gaast A, Dees J for the Rotterdam Oesophageal Tumour Study Group (1996) Cisplatin and etoposide in oesophageal cancer: a phase II study. *Br J Cancer* **74**: 980–984

Spiridonidis CH, Laufmann LR, Jones JJ, Gray DJ, Cho CC and Young DC (1996) A phase II evaluation of high dose cisplatin and etoposide in patients with advanced esophageal adenocarcinoma. *Cancer* **78**: 2070–2077

Stahl M, Wilke H, Meyer H-J, Preusser P, Berns T, Fink U, Achterrath W, Knipp H, Harstrick A, Berger M and Schmoll H-J (1994) 5-Fluorouracil, folinic acid, etoposide and cisplatin chemotherapy for locally advanced or metastatic carcinoma of the esophagus. *Eur J Cancer* **30A**: 325–328

© Cancer Research Campaign 1998